# The Use of a High Offset Fully Coated Collarless Cementless Stem Does Not Result in Early Failures in Total Hip Arthroplasty

**DOI:** 10.7759/cureus.45982

**Published:** 2023-09-26

**Authors:** Kiran R Madhvani, Matthew Hampton, Naren Garneti

**Affiliations:** 1 Trauma & Orthopaedics, Rotherham District General Hospital, Rotherham, GBR

**Keywords:** hip surgery, subsidence, corail, cementless stem, collarless stem, high offset, total hip arthroplasty

## Abstract

Introduction: The Corail femoral stem has excellent long-term survivorship in total hip arthroplasty (THA). However, there remains a paucity of information on the specific performance of the high offset collarless stem in relation to subsidence, loosening, offset, and failure rates.

Methods: Retrospective data were collected on all consecutive high offset collarless Corail stems implanted at a single centre in the UK. Data included patient demographics, femoral Dorr classification, radiographic analysis for radiolucent lines, and stem subsidence. The postoperative femoral offset was measured against the native offset of the contralateral hip. Any early failures, re-operations, or requirements for revision surgery were recorded.

Results: We identified 162 stems for inclusion in the study. Ninety-five patients were male. The mean age was 60.5 (40 to 78) years, and the mean BMI was 29.8 (21 to 50) kg/m^2^. The mean length of follow-up was 84.5 (12-130) months. Subsidence was recorded on 113 (69.7%) stems. The mean amount of total stem subsidence in the whole cohort was 1.62mm (0 to 3.9mm). There was no correlation between the amount of subsidence and the preoperative Dorr classification, age, sex, BMI, or indication for surgery. Radiolucent lines were exclusively seen in stems paired with a large-diameter 36-mm femoral head. The high offset stem accurately reproduced native femoral offset; the mean difference in offset was -1.21mm (-24mm to +21mm). There were no early failures, re-operations, or revision surgeries.

Conclusion: The use of a high offset stem can accurately reproduce native femoral offset when chosen for THA. The high offset collarless Corail stem does not result in early failures in THA, and we support its use.

## Introduction

Total hip arthroplasty (THA) is a well-established treatment for end-stage osteoarthritis (OA) of the hip. Cementless fixation of the femoral component allows for a strong biological fixation between the stem and bone, permitting the longevity of the implants and protecting against aseptic loosening [1]. One of the most widely used cementless stems is the fully hydroxyapatite-coated titanium stem, the Corail femoral stem (DePuy Synthes, Warsaw, Indiana, USA) [2].

The Corail stem has an excellent survivorship rate of 91.82% at 15 years on the National Joint Registry (NJR) for England, Wales, Northern Ireland, and the Isle of Man [2]. The Corail femoral stem traditionally has four main subtypes: standard offset with collar (KA), lateral offset with collar (KLA), standard offset without collar (KS), and high offset without collar (KHO). The KHO high offset collarless stem was introduced in 2014, and the KLA collared stem was renamed the 127° high offset collared stem in 2015.

There has been an increase in the use of KA collared Corail prostheses due to the perceived advantages it has in providing early axial and rotational stability, protection against early postoperative stem subsidence, and the dispersion of medial calcar forces, resulting in a reduced incidence of radiolucent lines [3-4]. Although the above effects are desirable, they were previously not supported by clinical studies or historical long-term survivorship data comparing collarless and collared stems [5-6]. However, studies published recently have supported the protective effects of a calcar collar and the superior performance of collared cementless femoral stems over collarless cementless femoral stems [7-8].

Currently, the NJR does not differentiate between each Corail stem sub-type in the annual report, and the majority of published data indicating the success of the Corail stem are based on the KA collared standard offset stem [9]. There is currently a paucity of data on the specific performance of the Corail high offset collarless stem, which aims to more accurately restore native femoral offset, soft tissue tensioning, and the preservation of femoral bone stock.

The primary aim of this study was to evaluate the early radiographic outcomes of the high offset collarless Corail cementless stem. We evaluated the stem size, the presence of radiolucent lines, stem subsidence, the ability of the stem to recreate the native femoral offset, and all-cause stem survivorship in a large consecutive cohort. We hypothesised that the KHO collarless high offset Corail stem would be better at reproducing the native femoral offset when appropriately sized and would not result in excessive stem subsidence or early implant failures.

## Materials and methods

**Figure 1:**
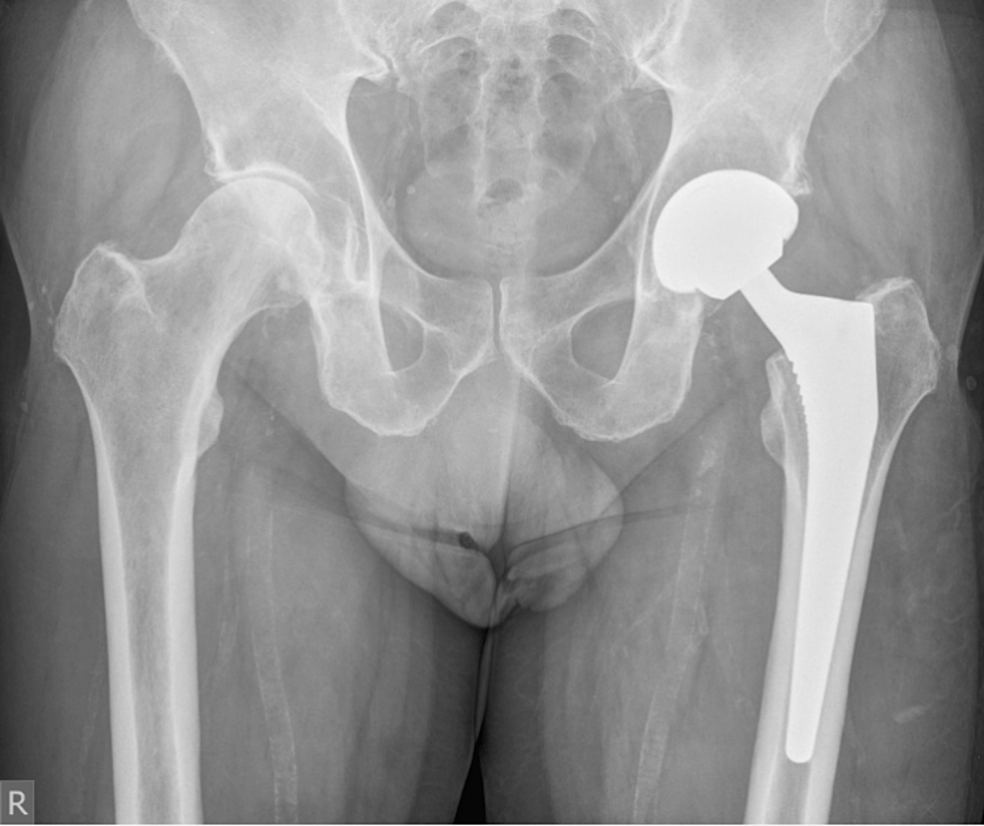
Standing anteroposterior radiograph showing a left high offset collarless Corail stem

A retrospective review of all high offset collarless Corail stems implanted over a 10-year period between March 2010 and March 2020 was performed (Figure 1).

All patients with high offset collarless Corail stems were identified from the senior authors’ prospectively completed database. Surgery was carried out either by the senior author or their Fellow or a specialist trainee, with the senior author as the first assistant. All surgery was performed at Rotherham District General Hospital, Rotherham, United Kingdom (UK).

A minimum of one-year follow-up was necessary for inclusion in the study to ensure an appropriate time period was available to accurately identify early stem subsidence on serial radiographs. All patients had complete data sets available for analysis.

All patients had equivalent standard anteroposterior (AP) pelvic radiographs taken both preoperatively and during subsequent postoperative follow-up periods. The focus film distance was 100cm, centred on the symphysis pubis, with the position of the lower limbs internally rotated by 15 degrees to demonstrate the greater trochanters in profile.

Patients’ demographics, BMI, and indications for surgery were recorded. Preoperative radiographs were used to classify the femora as per the Dorr classification [10].

Intraoperative data collected included femoral stem size, femoral head size, bearing surfaces used, and brand of acetabulum shell implanted.

Serial postoperative radiographs were analysed for stem subsidence and the presence of radiolucent lines. A comparison to the patient's native offset was made if a contralateral THA was not present. Any complications, re-operations, or requirements for revision surgery were recorded.

Stem subsidence was evaluated on serial postoperative radiographs using the Engh and Massin method [11], while postoperative femoral offset was compared with the contralateral native femoral offset using the Sundsvall method [12]. Judgment on the appropriate sizing of the stem was made on postoperative radiographs; any femoral stem with more than 1-2mm of cancellous bone between the prosthetic stem and the femoral cortex on the AP pelvic radiograph was considered an undersized stem. A gap of greater than 1-2mm at the prosthesis-femoral cortex interface was used to determine an undersized stem based on the operative technique recommended by the implant manufacturers.

Classification and radiographic measurements were performed by two independent orthopaedic surgeons. Any discrepancies were resolved by the senior author. All patients had radiographs available for analysis preoperatively and at day one, six weeks, six months, and one year postoperatively. Further radiographs were available at three (n=2), five (n = 89), and 10 years (n = 71) postoperatively for patients who had reached those follow-up points. The NJR data and hospital records were checked to evaluate the data on any stems that may have undergone revision surgery.

Statistical analysis 

The data were checked for normal distribution with the D'Agostino-Pearson normality test. The unpaired t-test was used to identify if there was a significant difference between groups. Spearman's correlation coefficient was calculated to determine the correlation between independent factors with both stem subsidence and radiolucent lines.

All analyses were completed on GraphPad Prism version 8.4.2 (GraphPad Software, San Diego, California, USA). Statistical significance was assumed at p<0.05.

Surgical technique

All THAs included in the study were implanted through a posterior approach. The acetabular shell used was almost exclusively the Pinnacle shell (DePuy Synthes, Warsaw, Indiana, USA), except in nine cases where a Gryption shell (DePuy Synthes, Warsaw, Indiana, USA) was used in patients when there was an intraoperative concern with the stability of the Pinnacle trial shell.

Bearing surfaces were ceramic-on-ceramic in all but two cases, which were ceramic-on-polyethylene.

Although preoperative templating allowed for a presumption of stem size, all stems were selected based on careful intraoperative surgical technique. The high offset Corail stem was selected for patients with a high neck-shaft valgus angle, based on clinical judgement, in order to recreate the native offset and hip centre. The femoral canal was prepared by increasing the size of the broach sequentially until an optimum fit was achieved with longitudinal and rotational stability. All patients were encouraged to fully weight-bear from day one post-surgery.

## Results

We identified 162 high offset Corail stems that were suitable for inclusion in the study. This included 95 male and 67 female patients. The mean age at the time of surgery was 60.5 (40 to 78) years, with a mean BMI of 29.8 (21 to 50) kg/m2. The mean length of follow-up in the cohort was 84.5 months (12-130 months).

The indication for surgery was OA of the hip in 124 (76.5%) patients; 22 (13.6%) patients had avascular necrosis (AVN) of the femoral head; nine (5.5%) patients underwent THA for a fractured neck of femur (NOF); six (3.7%) patients had sequelae of developmental dysplasia of the hip; and one (0.6%) patient had a non-union following internal fixation for a NOF fracture.

We classified 30 as type A femora, 99 as type B, and 33 as type C femora as per the Dorr classification.

There were five stems judged to be undersized on the postoperative radiographs, and the remaining 147 stems were considered to be appropriately sized.

Subsidence of the femoral stem was only seen early; we recorded the majority of subsidence on the six-week radiograph, with a small degree occurring up to the six-month radiograph. Stem subsidence wasn’t observed after six months postoperatively. A degree of subsidence was recorded on 113 (69.7%) stems. The mean amount of total stem subsidence in the whole cohort was 1.62mm (0 to 3.9mm). Subsidence was seen in all of the undersized stems, although due to the small number of undersized stems, we were unable to determine its significance.

**Figure 2:**
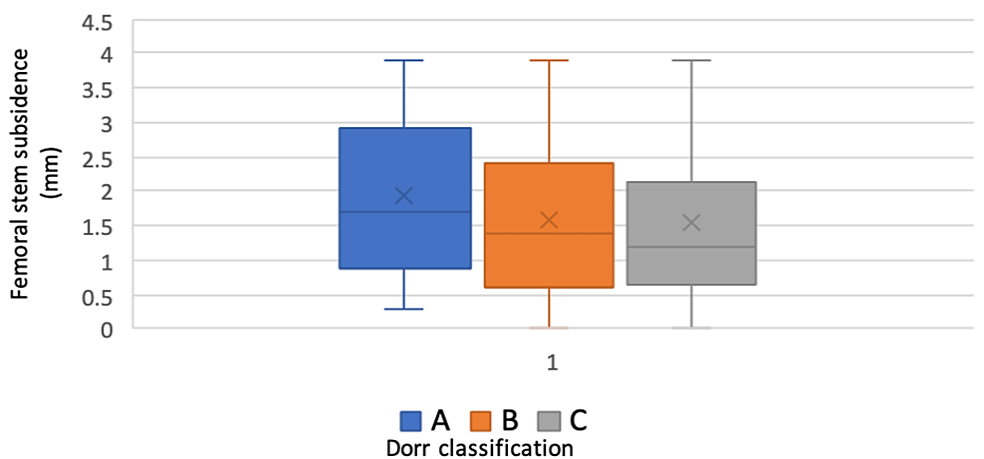
Box plot showing the amount of subsidence relative to the preoperative femoral Dorr classification mm: millimetres; A, B, or C: Dorr classification of femur

There was no significant difference identified between the amount of stem subsidence observed relative to the preoperative femoral Dorr classification (A=P:0.89, B=P:0.37, C=P:0.28) (Figure 2).

**Figure 3:**
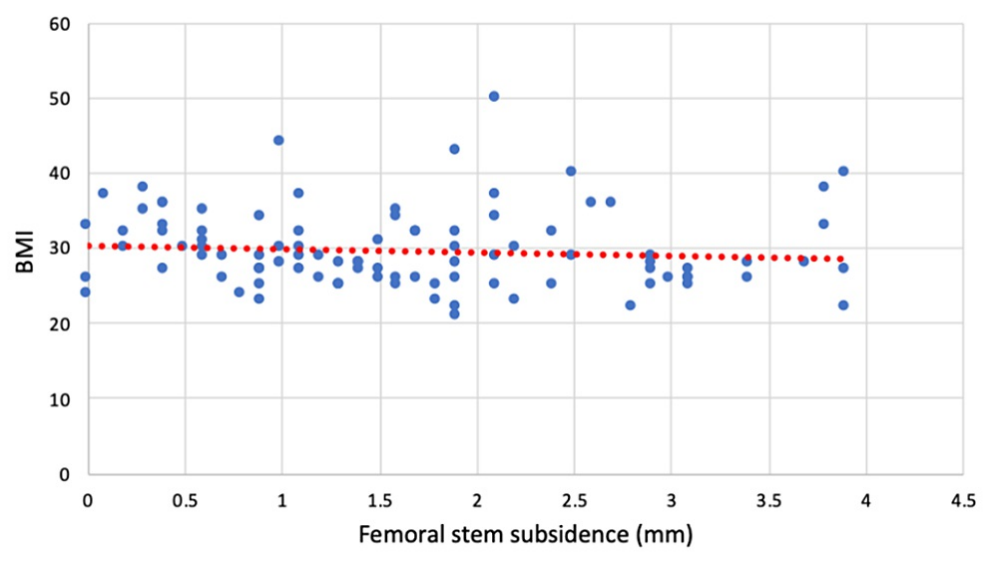
Scatter graph of measured subsidence in relation to patient BMI BMI: body mass index; mm: millimetres

Stem subsidence did not appear to be correlated to the patient's BMI at the time of surgery (rs =0.12), sex (rs =0.12), femoral head size, or indication for surgery (Figure 3).

Specifically, we did not see any significant subsidence in the stems used for patients with NOF fractures. However, it is important to emphasise that the nine patients treated for NOF fractures were all young with type A or type B femora. The senior author would typically use a cemented stem for older patients with NOF fractures.

**Figure 4:**
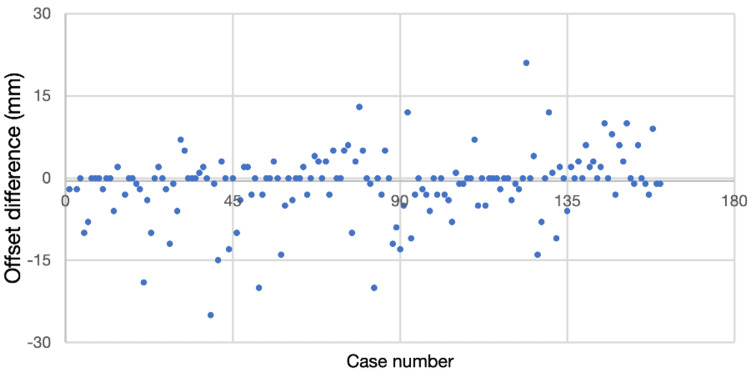
A scatter graph shows the offset difference in Corail high offset collarless stem from the contralateral hip for each case studied. mm: millimetres

When comparing the native femoral offset to the femoral offset created by the high offset collarless stem, the mean difference in offset was -1.21mm (-24mm to +21mm) (Figure 4).

Radiolucent lines around the stem were rarely seen at the one-year follow-up radiograph. They were observed in five (3%) stems, exclusively seen in zones one and seven. All stems that demonstrated radiolucent lines were combined with a 36-mm femoral head; no stems with 28- or 32-mm femoral heads showed any evidence of radiolucent lines. The radiolucent lines did not progress on the five-year radiographs and appear to have no effect on clinical outcomes or stem survival. In addition to radiolucent lines, we analysed the postoperative radiographs for evidence of calcar resorption. Calcar resorption wasn’t observed in any radiographs in this study.

No patients in the study cohort underwent any re-operations, and to date, no stems have required revision surgery.

## Discussion

This study of 162 consecutive femoral stems demonstrates that the KHO Corail high offset collarless stem does not result in excessive early stem subsidence and accurately recreates native offset in THA. Although there is excellent data on the Corail stem, there is currently a paucity of data available on this specific stem design [13]. Concerns regarding initial stem stability and the proposed risk of subsidence have led to an increased use of the collared prosthesis [3].

Our study required a minimum follow-up period of 12 months, with a mean follow-up of 84.5 months. Failure of cementless stems generally occurs early, with peri-prosthetic fractures, dislocations, and early migration of implants secondary to poor fixation being the main reasons for early revision [14-15]. Late revisions due to aseptic loosening are particularly uncommon [16]. It is therefore essential to report the early outcomes of cementless stems in detail.

Collarless stems are advantageous due to the more even surface loading of the stem, resulting in complete osseointegration [17]. Collared stems have a propensity for initial proximal stem loading with less force transmission distally, which could lead to stress shielding and poor integration of the distal stem [18].

The use of a collared stem relies on an accurate femoral neck cut and the seating of the collar on the femoral calcar. Poor calcar-collar contact could potentially increase the risk of proximal femoral stress shielding and iatrogenic fracture and result in leg length discrepancies [18-21]. 

The collarless designs are also dependent on meticulous surgical technique; the sizing of the femoral stem in our study was conclusively chosen based on intraoperative trailing. If a collarless stem is undersized, it is likely that early subsidence could become problematic. The precise amount of radiographic stem subsidence required to become clinically significant is unknown [5,22]. Less than 2mm of radiographic subsidence is likely to be insignificant and could well be within the normal limits of error for radiographic assessment [23]. The mean amount of subsidence seen with the KHO stem in our study was 1.62mm.

We didn’t observe any clinical consequences of stem subsidence in our study. Subsidence is only seen in the early postoperative period; this is supported by multiple studies, with subsidence rarely seen after six months [24-25]. This is due to the strong bond formed by osseointegration once the prosthetic stem has settled in the femoral canal. Neither BMI nor the Dorr femoral type resulted in increased stem subsidence and should not be considered a contraindication to using a collarless stem design. Early weight-bearing was encouraged in all patients. It is our belief that weight bearing only enhances the stability of an appropriately sized collarless femoral stem prosthesis and should not be restricted to routine, uncomplicated THA, regardless of stem design.

Magill et al. [26] reviewed a large database of Corail stems, observing the effect of undersizing. They report that undersizing of the stem results in increased radiolucent lines, and the use of a collared prosthesis could protect against this. In our study, we didn’t observe excessive amounts of radiolucent lines, but when we did, we also found that they tend to be present in Gruen zones one and seven. It is possible that our findings are due to the small number of stems found to be undersized, which further supports the importance of appropriate sizing when using the Corail implant [5].

Large femoral heads increase torque in retroversion; this could also theoretically explain why radiolucent lines were exclusively seen in stems coupled with large-diameter 36-mm femoral heads. Collared stems allow for greater early torsional stability when compared to collarless stems, which may be protective against this finding and may explain why previous studies have identified fewer radiolucent lines with collared stems [26]. However, head size had no bearing on the survival of the stem in our cohort, which is in line with previous reports [27-28]. 

It is important to consider the risk of early iatrogenic peri-prosthetic fractures when using any cementless stems. Lamb et al.'s [7] registry study with biomechanical validation showed collarless stems to have an almost five-fold increased relative risk of peri-prosthetic fracture when compared to collared stems. Although significant, we do not believe these findings should be extrapolated to all cementless stems and to all surgeons using collarless designs. In our cohort, we saw no peri-prosthetic fractures using this specific stem design, and with thorough preoperative planning combined with careful intraoperative trialling, this should not distress surgeons using collarless stems.

One interesting finding from this study was the ability of the high offset Corail stem to accurately recreate native femoral offset. The neck-shaft angle of standard stem designs varies across manufacturers. Insufficient femoral offset can result in instability, limping, abnormal abductor function, and gait, with an increase in the requirements for walking aids. To the best of our knowledge, no previous study has investigated the ability of the high offset Corail stem to recreate native femoral offset. We found that the high offset stem was very accurate in recreating offset, with the mean difference in offset being only -1.21mm. It is the senior author's opinion that the high offset stem is often more suitable than the standard offset stem in the predominantly Caucasian population that we treat in the UK. This study was not designed as a comparative study to the standard offset stem, so this is simply an observation within our study cohort.

This study does have limitations; the retrospective nature of the study means that not all variables can be controlled. We tried to decrease variables by limiting the study to a single surgeon within a single centre and including all consecutive stems for analysis. This study reports radiographic outcomes and stem survivorship; we have not included patient-reported outcome scores in our analysis. We have, however, addressed the aims of our study by showing that early failures are not a concern when using the high offset collarless cementless Corail stem design.

## Conclusions

In conclusion, this study demonstrates that the high offset collarless Corail stem does not result in early failures in THA, and we support its use. Furthermore, preoperative femoral Dorr classification, BMI, or early postoperative weight bearing do not affect early stem subsidence. In addition, it is important to accurately size collarless stems, as undersizing could result in stem subsidence, and this study demonstrates that the use of a high offset stem can accurately reproduce native femoral offset when chosen for THA.
